# Diet quality and a traditional dietary pattern predict lean mass in Australian women: Longitudinal data from the Geelong Osteoporosis Study

**DOI:** 10.1016/j.pmedr.2021.101316

**Published:** 2021-01-07

**Authors:** Jessica A. Davis, Mohammadreza Mohebbi, Fiona Collier, Amy Loughman, Nitin Shivappa, James R. Hébert, Julie A. Pasco, Felice N. Jacka

**Affiliations:** a Deakin University, IMPACT – the Institute for Mental and Physical Health and Clinical Translation, School of Medicine, Barwon Health, HERB Building, Level 3, 285-299 Ryrie St, Geelong, VIC 3220, Australia; b Deakin University, Faculty of Health, Biostatistics Unit, Building BC, Room BC4.121, 221 Burwood Highway, Geelong, Burwood, VIC 3125, Australia; c Geelong Centre for Emerging Infectious Diseases (GCEID), Barwon Health, HERB Building, Level 3, 285-299 Ryrie St, Geelong, VIC 3220, Australia; d Barwon Health, PO Box 281, Geelong, VIC 3220, Australia; e Cancer Prevention and Control Program, University of South Carolina, Discovery 1 Building, Suite 200, 915 Greene Street, Columbia, SC 29208, USA; f Department of Epidemiology and Biostatistics, Arnold School of Public Health, University of South Carolina, 921 Assembly Street, Columbia, SC 29208, USA; g Department of Nutrition, Connecting Health Innovations LLC, Discovery 1 Building, Suite 200, 915 Greene Street, Columbia, SC 29208, USA; h Department of Epidemiology and Preventive Medicine, Monash University, Prahran, 553 St Kilda Rd, Melbourne, VIC 3004, Australia; i Department of Medicine-Western Health, The University of Melbourne, St Albans, Furlong Rd, St Albans, VIC 3021, Australia; j Centre for Adolescent Health, Murdoch Children’s Research Institute, Flemington Road, Parkville, VIC 3052, Australia; k Black Dog Institute, Hospital Rd, Randwick, NSW 2031, Australia; l James Cook University, 1 James Cook Dr, Douglas, QLD 4811, Australia

**Keywords:** Diet quality, Dietary patterns, Muscle mass, Skeletal muscle index, Sarcopenia

## Abstract

Low muscle mass is associated with reduced independence and increased risk for falls and fractures. Identification of modifiable risk factors for low muscle mass is thus imperative. This study aimed to examine the longitudinal relationship between both diet quality and patterns and lean mass in Australian women.

Data from n = 494 participants of the Geelong Osteoporosis Study’s 10- and 15-year women’s follow-ups were used (conducted in 2004–08 and 2011–14, respectively), and participants were aged 21–89 years. Self-reported lifestyle and demographics were collected, and food frequency questionnaire data informed the dietary exposure variables: the Australian Recommended Food Score (ARFS); the Dietary Inflammatory Index (DII); and a posteriori dietary patterns. The outcome, Skeletal Muscle Index (SMI), was calculated from DXA-derived appendicular lean mass (ALM) relative to height (ALM kg/m^2^). Analyses employed Generalised Estimating Equations.

A higher ARFS score positively predicted SMI over 5-years, and adjustments for age and physical activity did not attenuate this relationship (B:0.044, (95%CI 0.004, 0.084) kg/m^2^). Following adjustment, both an anti-inflammatory diet (B:-0.034, (95%CI −0.070, −0.002) kg/m^2^) and a ‘traditional’ dietary pattern predicted higher SMI (B:0.081, (95%CI 0.004, 0.158) kg/m^2^). No other associations were observed.

Our study reinforces the importance of diet quality for healthy, aging muscle mass. Furthermore, a less inflammatory diet and a diet comprising a wide variety of plant and animal foods may be conducive to maintenance of muscle mass in women. Further studies investigating diet quality’s impact on various muscle health measures over longer time periods are warranted.

## Highlights

• The Australian Recommended Food Score predicted SMI over 5-years.
• A less inflammatory diet and a ‘traditional’ diet predicted SMI over 5-years.
• Neither a ‘plant-focused’ nor ‘western’ diet predicted SMI over 5-years.

## 1 Introduction

As a result of reductions in birth rates and increases in life expectancy, the proportion of Australians aged over 65 years has increased from 12.3% in 1999 to 15.9% in 2019 (Australian Bureau of Statistics, 2019)). Projections suggest a continuing trend, with 20% of Australians estimated to be aged over 65 by 2030 and a concomitant increase in the prevalence of health conditions associated with ageing (Australian Government Department of Health and Ageing, 2008).

Older age is generally accompanied by a decline in physical function, much of which is attributable to muscle health reductions. Low muscle mass, one important component of reduced muscle health, is associated with increased frailty, reduced independence, and increased fall and fracture risk in older populations (Balogun et al., 2017). Low muscle mass also may contribute to chronic systemic inflammation due to increased intramuscular adiposity observed in poor muscle health (Addison et al., 2014), and may influence the development, or increase the severity, of type 2 diabetes mellitus (T2DM) due to skeletal muscle’s vital role in glucose metabolism (Kim and Kim, 2020). In women, a longer average life span - with its associated disability - means a greater decline in muscle mass compared to men (Launer et al. (1994).

While increasing age may be the primary cause of muscle health decline, the rate and severity of its decline may be attributable to many modifiable lifestyle factors including physical activity (PA), sleep, smoking, and nutrition (Hughes et al., 2001, Aisbett et al., 2017, Lee and Choi, 2019, Pasco, 2019, Granic et al., 2019). Nutrient-focused research has identified a number of dietary factors associated with muscle mass, including energy intake, protein quantity, timing and animal or plant source, vitamin D, iron, calcium, sodium, potassium, magnesium and zinc (Batsis and Villareal, 2018, Bauer et al., 2013, Ko et al., 2015, van Dronkelaar et al., 2018). These findings provide insight into the potential biochemical mechanisms of the nutrient-muscle health relationship. However, a reductionist approach to the consideration of diet does not accurately distinguish between individual nutrient effects because of the inevitable interactions between foods and nutrients. A focus on overall dietary quality and patterns considers the complexities of nutrition while also providing findings that are applicable to real-world dietary recommendations.

Due to the multidimensional nature of diet and challenges of capturing dietary intake (Naska et al., 2017), utilising both *a priori* diet quality scores and *a posteriori* dietary pattern analysis may more comprehensively capture dietary behaviour. The Mediterranean Diet Score (MDS) has been commonly used as an *a priori* measure of diet quality in muscle health research (Kelaiditi et al., 2016, Chan et al., 2016, Talegawkar et al., 2012, da Silva et al., 2019, Zbeida et al., 2014). While the MDS is associated with reduced risk of several health conditions, it may not serve as the optimal diet quality measure in non-Mediterranean populations (Martínez-González et al., 2017). The Australian Recommended Food Score (ARFS) (Collins et al., 2008), however, is a diet quality index based on the Australian Dietary Guidelines and is therefore directly relevant to the Australian population. To date, no muscle health research has utilised the ARFS. The Dietary Inflammatory Index (DII®) (Shivappa et al., 2013), another measure of diet quality, quantifies the inflammatory potential of individuals’ diets and, due to the potential role of inflammation in sarcopenia, has been employed in muscle health research in two studies assessing adults and children (Cervo et al., 2020, Kwame Amakye et al., 2018).

While *a priori* dietary indices provide a pre-defined score measuring adherence, *a posteriori* dietary pattern analysis provides an indication of food consumption patterns within a given population. The relationship between dietary patterns and muscle mass has been investigated cross-sectionally, with no associations reported (Mohseni et al., 2017, Oh et al., 2014). There are no studies investigating either dietary patterns or the ARFS and muscle mass over time and DII use in muscle mass research is limited. The aim of the current study was therefore to examine the longitudinal relationship between both diet quality and patterns and skeletal muscle mass, measured in Australian women over a five-year period.

## 2 Methods

### 2.1 Participants

**Fig. 1:**
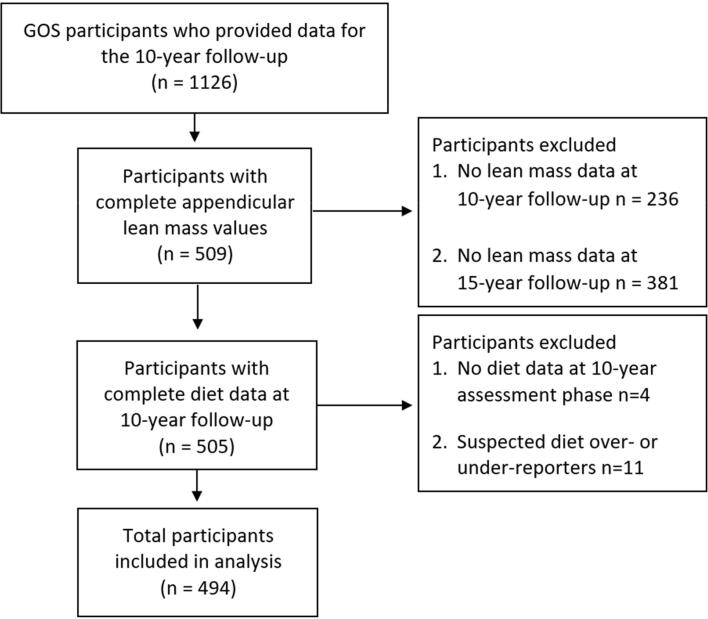
Flow diagram for GOS participant selection. 10-year and 15-year follow-ups were conducted in 2004–08 and 2011–14, respectively.

Participants comprised 494 women who provided complete dietary and appendicular lean mass data measured using dual x-ray absorptiometry (DXA) at both the 10- and 15-year follow-up assessment phases of the Geelong Osteoporosis Study (GOS) (Pasco et al., 2012) (Fig. 1). The GOS is an ongoing prospective cohort study that has been collecting women’s data since 1993. Participants were invited to participate via random selection from the electoral roll for the Barwon Statistical Division in south-eastern Australia, providing a sampling frame reflecting the various backgrounds of those in the region. Further details of recruitment, data collection, and participation rates have been published elsewhere (Pasco et al., 2012). In brief, n = 1494 participants were recruited at baseline and a further 246 women aged 20–29 years on the 2005 electoral roll were recruited in 2006–8 and added to the cohort; in total, n = 1126 and n = 848 completed the 10- and 15-year assessment phases, respectively. The GOS was approved by the Human Research Ethics Committee at Barwon Health and written, informed consent is collected from all participants.

## 3 Measures

### 3.1 Anthropometry and body composition

Height was measured using a wall-mounted stadiometer and recorded to the nearest 0.1 cm, and body composition data were collected from whole body DXA; Lunar DPX-L (10-year) and Lunar Prodigy-Pro (15-year), (LUNAR Corporation, Madison, WI, USA). DXA whole body fat (g) was utilised for the body fat measure, and appendicular lean mass (ALM; sum of lean mass for arms and legs) was identified as a proxy measure for total appendicular muscle mass, from which the primary outcome variable, skeletal muscle index (SMI), was calculated.

### 3.2 Skeletal muscle mass cut-off point

SMI was defined as ALM/height^2^ equal to kg/m^2^. As per recommendations from the recently revised European Working Group on Sarcopenia in Older People (EWGSOP2) (Cruz-Jentoft et al., 2019), the cut-off for low SMI was specified as 5.5 kg/m^2^. Furthermore, the values on which this recommendation were originally established were from a sample from the GOS comprising, amongst others, women from the same 10-year follow-up, making the validity of this cut-off directly transferable to the current sample (Gould et al., 2014).

### 3.3 Dietary data

At both 10- and 15-year timepoints, the Australian Cancer Council’s Dietary Questionnaire for Epidemiological Studies (DQES) was employed for the collection of dietary data. This questionnaire has been validated in a similar population (Giles and Ireland, 1996). The DQES collects information on quantity and frequency of consumption of 74 foods and six alcoholic beverages in the preceding 12 months.

#### 3.3.1 Diet diet quality scores

From the DQES data, the ARFS and DII were calculated. The ARFS provides a score indicating the degree of adherence to the Australian Dietary Guidelines by allocating a score each for (i) vegetables, nuts and beans (ii) fruit (iii) protein containing foods (iv) grains (v) dairy (vi) fats, and (vii) alcohol, which are totalled for participants’ ARFS, with scores ranging from 0 to 74 (Collins et al., 2015). The DII provides a score reflecting the level of inflammatory potential of the diet; a positive value is considered pro-inflammatory, while a negative value is anti-inflammatory. Scores range from a theoretical low of −8.87 to +7.98 (Shivappa et al., 2013). The original DII comprises 45 food components that have been associated with either systemic pro-inflammation or anti-inflammation, however the DQES provides data on only 22 of these food components. Therefore, the DII utilised herein has been modified by the original authors of the DII (Shivappa et al., 2013). The DII and ARFS have both been validated in similar populations (Collins et al., 2015, Tabung et al., 2015).

#### 3.3.2 Dietary patterns

*A posteriori* dietary patterns were previously generated for this cohort from the DQES data by employing a principle component analysis (PCA) using orthogonal varimax rotation (Jacka et al., 2010). From the 74 original DQES items, those with a loading of less than |0.2| on all factors were excluded, resulting in 68 food items informing the final dietary patterns.

#### 3.3.3 Other potential confounders

Lifestyle and demographic variables were collected at both 10- and 15-year follow-up assessment phases, including age, DXA whole body fat mass (g), and area-based socio-economic status (SES), measured by the Index of Relative Socio-economic Advantage and Disadvantage (IRSAD). The IRSAD takes social and economic circumstance into account for both participants and their households, and is considered a measure of both relative advantage and disadvantage (Australian Bureau of Statistics., 2033). Early-life SES has previously been reported to predict muscle strength in later life, with a particular emphasis on women (Cheval et al., 2018). This association is potentially explained by chronic stress related to income, education, and unhealthy behaviours (Cheval et al., 2018). In addition, self-reported lifestyle and demographic variables included highest level of education, marital status, PA (dichotomised into active (comprising “very active” or “active”) or not active (comprising “sedentary”, “limited”, “inactive”, “chair/bed-ridden”, or “bedfast”)), current smoking, average hours sleep, total dietary energy kJ/d, and total dietary protein g/d.

Current medication use (which may impact muscle health) was self-reported and dichotomised into yes/no for each category queried. These included: i) Non-steroidal anti-inflammatory drugs, anti-rheumatoid agents, rubefacients, or topical analgesics; ii) Adrenal steroid and gonadal hormones, anabolic agents, insulin, or hypoglycaemic agents; and iii) Hormonal contraception. Self-reported medical conditions that may affect muscle health also were dichotomised into yes/no and included rheumatoid arthritis, thyroid conditions, multiple sclerosis, muscle weakness, or pernicious anaemia. Presence of diabetes was identified as fasting plasma glucose <7.0 mmol/L, use of anti-hyperglyacemic agents, and/or self-reported diabetes, and clinical records were used to distinguish between Type 1 Diabetes Mellitus (T1DM) and T2DM.

#### 3.3.4 Statistical analyses

Mean and median sample characteristics were extracted for parametric and non-parametric variables, respectively. Data were cleaned, outliers identified, and skewness assessed with descriptive statistics, graphical displays, and Shapiro–Wilk test for normality, respectively.

Due to the longitudinal structure of the outcome and time-updating nature of the dietary exposures at 10- and 15-year timepoints, Generalised Estimating Equation (GEE) models were utilised. The GEEs assumed continuous normally distributed outcome (SMI) with an identity link function. A robust variance estimator was used for estimating the models’ standard errors, utilising an unstructured covariance pattern to account for within participants’ autocorrelation across timepoints. The GEE models included a nominal factor for measurement time (10- to 15-year follow-up), time-updated diet measurement as the exposure of interest, and a time by diet two-way interaction. The time-by-diet interaction term examined the relationship between diet change (from 10- to 15-year follow-up) and SMI.

In order to allow for comparison between the varying scales, the three dietary exposures (ARFS, DII, and dietary patterns) were converted to z-scores.

To evaluate potential confounders, pairwise correlations between the potential confounder and both outcome and exposure were investigated, followed by a trivariate GEE for each of the *a priori* identified confounders*.* For the variables that significantly altered the B coefficient of the main effect by more than 10% in the trivariate model, a backwards stepwise method identified confounders included in the final model, with significance set at p < 0.05. In addition, confounders based on previous literature including age, PA, smoking, dietary protein, and dietary energy were also tested and, irrespective of their significance, included in the final models on the basis of their well-established relationships with both diet and SMI (Bloom et al., 2018). Interaction terms for each of the dietary exposures and confounders were also examined.

An exploratory analysis investigated the effect modification potential of hormone therapy (HT) by implementing a GEE model and including HT as a nominal factor and HT by DII two-way interaction; marginal B coefficients of the DII for the DII by HT interaction term was reported. An exploratory analysis was also conducted to further explore possible effect modification by age. The median age of 50.3 years served as the cut-off, which is supported by previous literature where significant decreases in women’s muscle mass were identified in the fifth decade of life (Kallman et al., (1990)).

All analyses were conducted using Stata 16.0 (StataCorp LP. College Station, TX, USA) and the analysis plan was preregistered at Open Science Framework (https://osf.io/wxzpv).

## 4 Results

Participants with DXA lean mass data at the 10-year follow-up who were lost to follow-up were slightly older, with a mean SMI similar to the current sample; however, twice as many were classified as having a low SMI compared to the study sample.

Study sample characteristics for 10- and 15-year follow-ups are summarised in Table 1. At 10-year, participants were aged 21 to 89 years, over half of the sample had completed education beyond secondary school, and the majority was considered physically active. One-third of participants were currently using medications that may have an impact on muscle health. Both the mean ARFS and the median DII scores were slightly below the midpoint and pro-inflammatory according to the definition of each dietary score, respectively. At follow-up a greater percentage of participants was inactive, had medical conditions which may impact muscle health, and had a low SMI. Mean total body fat also increased over this time period.

**Table 1:** Participants characteristics at GOS 10- and 15-year follow-ups.

	10-year (n = 494)	15-year
Age, years, mean (±SD)	50.3 (16.0)	
Education, n (%)*		
• Primary or some secondary school	215 (43)	217 (44.2)
• Completed secondary or vocational training n (%)	127 (26.5)	128 (26.1)
• Tertiary education	146 (30)	146 (29.7)
Marital status, n (%)^^^		
• Single	32 (6.5)	32 (6.5)
• Married	317 (64.6)	319 (64.6)
• de facto	35 (7.1)	35 (7.1)
• Separated/divorced	51 (10.4)	52 (10.5)
• Widowed	56 (11.4)	56 (11.3)
IRSAD score n (%)^^^		
• 1	75 (15.2)	76 (15.4)
• 2	99 (20.1)	100 (20.2)
• 3	109 (22.2)	110 (22.2)
• 4	99 (20.1)	99 (20.0)
• 5	110 (22.4)	110 (22.2)
BMI, mean (±SD)	26.0 (4.5)	26.9 (4.9)
Body fat (kg), median (IQR)	37.8 (32.4–43.0)	50.8 (34.6–46.2)
SMI, mean (±SD)	6.5 (0.6)	6.5 (0.7)
• Low SMI < 5.5 kg/m^2^, n (%)	22 (4.5)	34 (6.8)
• High SMI > 5.5 kg/m^2^, n (%)	472 (95.5)	460 (93.2)
Physical activity#		
• Inactive, n (%)	82 (16.6)	130 (26.8)
• Active, n (%)	412 (83.4)	355 (73.2)
Current smoker, n (%)	56 (11.3)	46 (9.4)
Current use of medications, n (%)	150 (30.4)	149 (30)
Medical conditions, n (%)	65 (13.2)	82 (16.5)
ARFS, mean (±SD)	33.7 (9.0)	33.4 (8.8)
DII, median (IQR)	0.9 (0.0–1.6)	0.6 (0.2–1.6)
Total daily energy (kJ), median (IQR)	6,120.5 (4,962–7,613)	5674.1 (4588, 7386)
Total daily protein (g), median (IQR)	72.27 (57.06, 87.93)	68.76 (53.96, 85.84)
Sleep (hours/night)	6.9 (1.3)	6.8 (1.3)

IRSAD = The Index of Relative Socio-economic Advantage and Disadvantage, lower scores indicative of greater disadvantage and lack of advantage, BMI = Body Mass Index, SMI = Skeletal Muscle Index, *n = 6 missing data, ^#^n = 9 missing data, ^^^n = 3 missing data

10-year and 15-year follow-ups were conducted in 2004–08 and 2011–14, respectively.

### 4.1 Diet quality scores and dietary patterns

Prior to standardisation, diet quality score ranges for the AGRF and DII were 5 to 60 and 2.67 to + 3.03, respectively.

Three *a posteriori* dietary patterns were identified from the PCA and named: i) plant-focused, ii) western, and iii) traditional. The plant-focused pattern was characterised by positive factor loadings on fruits, vegetables, legumes, fish, nuts, rice, tofu, yogurt, red wine, and eggs, and negative loadings on white bread. The western pattern included positive factor loadings on pizza, potato chips, processed meats (sausage, meat pies, salami, bacon), tomato sauce and pasta, and negative loadings on fruits. The traditional pattern was characterised by positive loadings on vegetables, jam, tinned fruit, red meat (beef and lamb), biscuits, ice cream, fish, and high-fibre cereals, with no negative loadings (Jacka et al., 2010).

### 4.2 Identification of potential confounders

Confounders identified in trivariate models included current use of HT for the DII, and historical use of HT for the dietary patterns. HT, age, PA, smoking, protein, and energy were further tested as two-way interactions with both diet and follow-up period.

### 4.3 The predictive value of diet on SMI over five years

Results from the GEEs assessing the relationship of the ARFS, DII (diet quality scores) and the three dietary patterns to SMI are presented in Table 2. The ARFS positively predicted SMI over the 5-year period with an increase of 0.044 kg/m^2^ of SMI for one ARFS point. Adjustment for age, PA, age, smoking, protein, and energy did not affect these results.

**Table 2:** Predictive values of the ARFS, DII, and dietary patterns and skeletal muscle index over five years.

Diet quality scores	B	95% CI	p value
**Australian Recommended Food Score**			
Model 1	0.044	0.004, 0.084	0.030**
Model 2^^^	0.045	0.003, 0.090	0.038**
**Dietary Inflammatory Index**			
Model 1	−0.019	−0.051, 0.014	0.26
Model 2^#^	−0.034	−0.070, 0.002	0.07
**Dietary patterns**	**B**	**95% CI**	**p value**
**Plant-focused**			
Model 1	0.025	−0.026, 0.076	0.34
Model 2^#^	0.013	−0.054, 0.080	0.71
**Western**			
Model 1	−0.002	−0.061, 0.571	0.95
Model 2^#^	−0.028	−0.131, 0.076	0.60
**Traditional**			
Model 1	0.051	−0.003, 0.105	0.07
Model 2^#^	0.081	0.004, 0.158	0.039**

Model 1 unadjusted, ^^^Model adjusted for age, physical activity, smoking, dietary protein, dietary energy, ^#^Model adjusted for age, physical activity, smoking, dietary protein, dietary energy, and hormone therapy.

**p < 0.05.

10-year and 15-year follow-ups were conducted in 2004–08 and 2011–14, respectively.

The DII was not predictive of SMI in the unadjusted model, however B coefficient size and significance increased with adjustment, suggesting negative confounding by age, PA, smoking, protein, energy, and current hormone therapy. The B coefficient for both models of the DII was negative, suggesting an anti-inflammatory diet associated with increases in SMI.

Similarly, the traditional dietary pattern suggested a trend in the unadjusted model, and B coefficient size and significance increased following adjustment to 0.081 kg/m^2^ of SMI for each point of the traditional dietary pattern.

Two-way interactions were explored between all dietary exposures and age, PA, smoking, HT, protein, and energy, and between age and PA. An interaction between the DII and current use of HT was observed. In women who were currently taking HT (n = 48), there were no associations observed between the DII and SMI; however, in those not taking HT (n = 883) there was an inverse association (marginal B −0.042, (95%CI −0.080, −0.005) kg/m^2^). No other two-way interactions were apparent.

The effect modification potential of age (<50.6 years and ≥50.6 years) was explored to assess whether the impact of diet on SMI varies between younger and older participants. No associations between diet and SMI were observed in the older group (≥50.6 years), however, in the younger group (<50.6 years) the traditional dietary pattern positively predicted SMI (marginal B 0.090, (95%CI 0.010, 0.170)kg/m^2^). The magnitude of this association was further strengthened with adjustment (marginal B 0.147, (95%CI 0.047, 0.248)kg/m^2^) to 0.147 kg/m^2^ of SMI per point increase of the traditional dietary pattern. No associations between other dietary exposures and SMI were evident in the younger group.

## 5 Discussion

A longitudinal association was observed between measures of dietary quality and SMI over 5 years. While our study is the first to investigate longitudinal associations between dietary patterns and muscle mass, previous studies reported no association between dietary patterns and muscle function or sarcopenia over five and four years, respectively (Granic et al., 2016, Chan et al., 2016). Similarly, previous literature reports conflicting findings of the longitudinal relationship between diet quality and muscle mass in women, both of which utilised the Baltic Sea Diet Score over three and ten years (Perälä et al., 2017, Isanejad et al., 2018). Although researchers reported significant associations over three years, in the ten-year study Perala et al. reported positive associations between diet quality and muscle strength, but not lean mass. These findings may be attributable to declines in age-related muscle strength preceding muscle mass loss (McGregor et al., 2014). Moreover, the study’s mean age was 60 years, which may have been too young to observe significant changes in muscle mass, and similar findings to Perala at al. have been reported in literature with lower mean ages (Mohseni et al., 2017, Kelaiditi et al., 2016, Welch et al., 2013).

While neither a plant-focused nor a western dietary pattern predicted SMI, the traditional dietary pattern identified as a predictor of SMI is considered a healthy “Anglo-Australian” pattern, consisting of a variety of animal proteins, fibrous vegetables, and wholegrain cereals. Components of this dietary pattern have been associated with reduced systemic inflammation (Munch Roager et al., 2019, Zhu et al., 2018), which may be attributable to micronutrients and fibre. Furthermore, it is well-established that protein benefits muscle health and that anabolic resistance increases protein requirements. With greater bioavailability of animal compared to plant protein (Bauer et al., 2013, van Vliet et al., 2015) our findings may reflect the traditional dietary pattern’s nutritional variety, in contrast to the plant-focused and western patterns, which are lower in animal and plant foods respectively. Diet variety also may explain our findings of the ARFS predicting SMI over five years. As the ARFS measures adherence to the Australian Dietary Guidelines, the score takes into account all food groups and may therefore provide a proxy measure of variety. Indeed, reduced nutritional variety has been associated with sarcopenia (Lim, 2020, Tanimoto et al., 2013) and, in conjunction with low SMI, longer hospitalisations (Lo et al., 2017). Also, frail American women with higher diet variety had greater muscle mass than those with lower variety (Bernstein et al., 2002). Greater nutritional variety may provide a wider array of nutrients important for muscle health, which may become more important with the physiological changes co-occurring with age, including reduced nutrient absorption, slowed metabolism, and anabolic resistance (Lim, 2020, van Vliet et al., 2015, Lovat, 1996, Hodson et al., 2019).

Our exploratory analysis suggested that an anti-inflammatory diet was associated with SMI in women who were not taking HT, but not in those who were. Whilst acknowledging the potential for this finding to be due to the smaller sample size in the HT group and reduced statistical power, this finding may reflect a mediating effect of HT on inflammatory mechanisms. Indeed, previous literature investigating HT’s effect on metabolic and vascular inflammatory markers has suggested an upregulation and downregulation by HT, respectively (Lo et al., 2017, Tanimoto et al., 2013, Cooper et al., 2007, Goudev et al., 2002). HT may therefore attenuate any anti-inflammatory potential of an anti-inflammatory diet. In addition, residual confounding may also exist in participants taking HT for menopause, which involves a host of endocrine changes potentially affecting metabolism and muscle pathways (Buckler, 2005).

Additional exploratory analyses of younger and older participants revealed that the traditional dietary pattern significantly predicted SMI over five years in women aged <50 years, but not in those aged ≥50 years. Furthermore, the magnitude of this association was the greatest observed in the current study. These findings may reflect the importance of establishing diverse dietary habits in early life for the development of healthy muscle mass prior to age-related atrophy. However, these findings may also be an artefact of power issues, with fewer younger participants eating a traditional dietary pattern compared to older participants (data not shown).

Previous literature forming the basis for clinical SMI cut-off values identified T-scores based on standard deviations of 0.77 kg/m2 (Gould et al., 2014). Our findings suggest that an increase of one unit in diet quality index or dietary pattern may result in increases of 0.034–0.147 kg/m^2^ of SMI. This may therefore suggest that modest increases in diet quality may positively contribute to SMI.

## 6 Strengths and limitations

The slightly differential associations detected between the ARFS compared to the DII may be attributable to the methods used to derive the scores. The ARFS was developed to be extracted directly from the DQES used in this study, and therefore all parameters in the algorithm were available for analysis. In contrast, the DII calculated for the current study included 22 of the 45 food components identified in the original DII. Furthermore, while the range of ARFS scores in the current sample was quite wide (5–60 of a possible 0–74), the range of DII scores was limited (−2.67 to +3.03 of a possible −8.87 to +7.98). Diets of the current sample participants may therefore not have provided a range wide enough to indicate pro- or anti-inflammatory diets which have been observed in other musculoskeletal studies utilising the DII (Shivappa et al., 2018 Jan 1, Orchard et al., 2017).

Moreover, the number of exposure variables examined means that there is the possibility our findings arose from type 1 errors. However, the findings of associations across different dietary measures lends credence to the presence of true relationships. A major issue with dietary assessments is the large measurement error associated with them; such measurement error can affect the ability to detect associations, also giving rise to the possibility of type 2 errors. In addition, food frequency questionnaires are subject to recall bias and contain only specific foods which may not capture details of participants’ diets. Moreover, our PA measure was based on a single question measure of mobility, which was insufficient to capture nuances in participants’ day-to-day activity. Future research addressing muscle health would benefit from utilising a robust measure of PA that captures work and leisure-time PA, and structured exercise. Strengths of our study lie in the large, well-characterised population-based sample of women with detailed measures of dietary intakes and an objective measure of lean mass. Furthermore, the GOS data allowed us to account for several potential confounding variables, and the follow-up assessments provided longitudinal data.

## 7 Conclusion

Our study reinforces the importance of diet quality in healthy ageing, with the ARFS, the DII, and a traditional dietary pattern all associating with SMI over five-years. Future research would benefit from well-controlled, long-term, prospective observational studies, as well as intervention studies aiming to assess the impact of improving diet quality on muscle health.

## Disclosure statement

Jessica A Davis is supported by an Australian Government Research Training Program Scholarship. Amy Loughman is supported by Deakin Dean’s Postdoctoral Research Fellowship. Dr. James R. Hébert owns controlling interest in Connecting Health Innovations LLC (CHI), a company that has licensed the right to his invention of the dietary inflammatory index (DII®) from the University of South Carolina in order to develop computer and smart phone applications for patient counseling and dietary intervention in clinical settings. Dr. Nitin Shivappa is an employee of CHI. The subject matter of this paper will not have any direct bearing on that work, nor has that activity exerted any influence on this project. Felice Jacka has received: (1) competitive Grant/Research support from the Brain and Behaviour Research Institute, the National Health and Medical Research Council (NHMRC), Australian Rotary Health, the Geelong Medical Research Foundation, the Ian Potter Foundation, The University of Melbourne; (2) industry support for research from Meat and Livestock Australia, Woolworths Limited, the A2 Milk Company, Be Fit Foods; (3) philanthropic support from the Fernwood Foundation, Wilson Foundation, the JTM Foundation, the Serp Hills Foundation, the Roberts Family Foundation, the Waterloo Foundation  and; (4) travel support and speakers honoraria from Sanofi-Synthelabo, Janssen Cilag, Servier, Pfizer, Network Nutrition, Angelini Farmaceutica, Eli Lilly and Metagenics. Felice Jacka has written two books for commercial publication. Julie A Pasco has received speaker fees from Amgen, Eli Lilly and Sanofi-Aventis and funding from the NHMRC and MRFF, Barwon Health, Deakin University, Australia and New Zealand Bone and Mineral Society (ANZBMS), Amgen-GSK OA-ANZBMS, Amgen Australia, the BUPA Foundation, Osteoporosis Australia, the Geelong Community Foundation, the Western Alliance, the Norman Beischer Foundation and the Victorian COVID-19 Research Fund. Mohammadreza Mohebbi has received Grant/research support from NHMRC, Deakin University School of Medicine, Deakin Biostatistics Unit, Institute for Mental and Physical Health and Clinical Translation, and Medibank Health Research Fund. Fiona Collier has no disclosures to report.

## CRediT authorship contribution statement

**Jessica A. Davis:** Software, Formal analysis, Investigation, Data curation, Writing - original draft, Writing - review & editing, Project administration. **Mohammadreza Mohebbi:** Methodology, Software, Formal analysis, Data curation, Writing - review & editing, Supervision. **Fiona Collier:** Writing - review & editing, Supervision. **Amy Loughman:** Writing - review & editing, Supervision. **Nitin Shivappa:** Software. **James R. Hébert:** Software, Writing - review & editing. **Julie A. Pasco:** Methodology, Resources, Data curation, Writing - review & editing, Supervision. **Felice N. Jacka:** Methodology, Investigation, Writing - review & editing, Supervision.

## Declaration of Competing Interest

The authors declare that they have no known competing financial interests or personal relationships that could have appeared to influence the work reported in this paper.
